# Who has access to counseling and testing and anti-retroviral therapy in Malawi – an equity analysis

**DOI:** 10.1186/1475-9276-8-13

**Published:** 2009-05-05

**Authors:** Ireen Makwiza, Lot Nyirenda, Grace Bongololo, Talumba Banda, Rhehab Chimzizi, Sally Theobald

**Affiliations:** 1 Research for Equity and Community Health Trust, P.O. Box 1597, Lilongwe, Malawi; 2 National Tuberculosis Control Programme, Ministry of Health, Malawi; 3 Liverpool School of Tropical Medicine, Pembroke Place, Liverpool, L3 5QA, UK

## Abstract

**Background:**

The HIV and AIDS epidemic in Malawi poses multiple challenges from an equity perspective. It is estimated that 12% of Malawians are living with HIV or AIDS among the 15-49 age group. This paper synthesises available information to bring an equity lens on Counselling and Testing (CT) and Antiretroviral Therapy (ART) policy, practice and provision in Malawi.

**Methods:**

A synthesis of a wide range of published and unpublished reports and studies using a variety of methodological approaches was undertaken. The analysis and recommendations were developed, through consultation with key stakeholders in Malawi.

**Findings:**

At the policy level Malawi is unique in having an equity in access to ART policy, and equity considerations are also included in key CT documents. The number of people accessing CT has increased considerably from 149,540 in 2002 to 482,364 in 2005. There is urban bias in provision of CT and more women than men access CT. ART has been provided free since June 2004 and scale up of ART provision is gathering pace. By end December 2006, there were 85,168 patients who had ever started on ART in both the public and private health sector, 39% of the patients were male while 61% were female. The majority of patients were adults, and 7% were children, aged 14 years or below. Despite free ART services, patients, especially poor rural patients face significant barriers in access and adherence to services. There are missed opportunities in strengthening integration between CT and ART and TB, Sexually Transmitted Infections (STI) and maternal health services.

**Conclusion:**

To promote equitable access for CT and ART in Malawi there is need to further invest in human resources for health, and seize opportunities to integrate CT and ART services with tuberculosis, sexually transmitted infections and maternal health services. This should not only promote access to services but also ensure that resources available for CT and ART strengthen rather than undermine the provision of the essential health package in Malawi. Ongoing equity analysis of services is important in analyzing which groups are unrepresented in services and developing initiatives to address these. Creative models of decentralization, whilst maintaining quality of services are needed to further enhance access of poor rural women, men, girls and boys.

## Background

Malawi is a small landlocked country in sub-Saharan Africa with a population of about 11 million people. 65% of the Malawian population is poor [1]. Health indicators remain poor, with maternal mortality rate being estimated at 1120 per 100,000 live births and infant mortality per 100,000 live births is 104 [2]. The country has been severely hit by the HIV and AIDS epidemic, with 12% of the adult population living with HIV or AIDS [3]. AIDS is the leading cause of death amongst the 15–49 year old age group [3]. The coming of Anti-retroviral therapy (ART) has brought optimism amongst Malawians through improving the quality of life of those living with the virus.

However, due to financial, infrastructural and human resource constraints not all of those in need of ART are reached by the drugs, leaving an enormous short fall [4,5]. This means that difficult choices have to be made about the where and how of ART provision as this will have wide reaching health, social and economic consequences [6]. Promoting equity in health means addressing differences in health that are judged to be unnecessary, avoidable and unfair. 'These differences relate to disparities across socio-economic status, gender, age, social groups, rural/urban residence and geographical region' [7]. Studies on equity in access in health services in Malawi have shown that there are widening inequities among the rich and the poor and that interventions aimed at reducing inequities amongst the poor are not producing the expected outcomes [8]. Tuberculosis case detection is low amongst the poorest men and women with the poor spending more than twice their monthly income on the costs of accessing a TB diagnosis [9].

From an equity perspective, ART provision should not exclude certain population groups such as the rural populations, the poor and marginalized [10]. Given that health systems themselves are largely inequitable [10,11] promoting equity in ART is challenging. Equity needs to be assessed from multiple perspectives and not only seen through access or uptake of ART but analysed throughout the ART pathway and include adherence and treatment outcomes. In addition there is need to focus on the how and where Counselling and Testing (CT) services are provided and who is accessing them; given that CT is the entry point to ART access. There is also a need to assess the impact of HIV and AIDS service provision on the delivery and quality of other essential health services in Malawi. For example are the resources being made available for ART scale up serving to strengthen or undermine the delivery of the Malawian Essential Health Package? This paper synthesises available information to bring an equity lens on Counselling and Testing (CT) and Antiretroviral Therapy (ART) policy, practice and provision in Malawi. The evidence will be useful in assessing if ART implementation is following the policy principles of equitable access to ART in Malawi and form the basis for recommendations for policy and practice.

## Methods

A meeting was first held with the research team which agreed on the key priority areas to be considered in the data collation and analysis and identified the available sources of information. A search for published and unpublished literature and programme and monitoring reports was undertaken. Collation and analysis of pre-existing information and indicators from different Malawian stakeholders, such as the Ministry of Health (MoH), National AIDS Commission (NAC), and within Research for Equity and Community Health (REACH) Trust, and some key providers of CT and ART was undertaken. There is a growing number of published and unpublished reports on CT and ART in and from Malawi produced by different organizations such as MoH, Non-governmental Organisations (NGO) and research groups. We did not have a strict inclusion and exclusion criteria in selection and collation of reports, but included all those that contained information that could illuminate the debate on equity and CT and ART. We contacted authors for clarification in cases where data was unclear or hard to interpret.

The following box highlights some of the key challenges we faced in equity analysis of HIV prevalence, CT and ART data.

i. It was difficult to use the prevalence rates estimated by the National AIDS Commission as they do not include children or people over 49 years.

ii. Most data on ART access does not include detailed information on access by socio-economic groups, poverty status, or age (with the exception of adult or child classification).

iii. The CT data is not disaggregated by age and data disaggregated by sex is only available for some of the client groups.

Informal key informant interviews were conducted with key stakeholders from the Ministry of Health and the National AIDS Commission to supplement and triangulate the literature and data collected. The analysis and recommendations were discussed with key stakeholders from the Ministry of Health and the Department for International Development (DFID), Malawi. The data was supplemented by insights and quotations from a qualitative research project conducted by REACH Trust in Thyolo district which was aimed at exploring factors that influence access and adherence to ART. These insights are used to help explain and contextualize some of the findings. The study employed in-depth interviews and focus group discussions with patients on ART and those who had dropped out from ART.

## Results and discussion

### Introduction

This section explores the extent to which equity is mainstreamed in policies and guidelines for CT and ART provision. This is followed by an overview of the national picture on access to CT and ART and a synthesis of what is known about access by gender, age, geography and socio-economic status. In the last section three intersecting key barriers to promoting equity in CT and ART services are presented.

### CT and ART Policies and Guidelines: An equity analysis

Equity issues addressed through the CT policy and guidelines for CT provision include the promotion and provision of high quality, cost effective and accessible services which are youth friendly and accessible to vulnerable groups [12]. In addition, the government of Malawi commits to progressively provide access to affordable, high quality ART, to patients tested HIV positive and medically deemed in need of drug therapy [12]. Key stakeholders in Malawi realised the need for equity in access to treatment and have been active in promoting equity in ART roll out and this has been consolidated in the development of an Equity in access to ART policy. Based on this policy ART is provided free of charge on a first come-first serve basis [13]. Within this approach, the underlying principle of the ART programme is that each and every social group should be able to access drugs [14]. Specific identification of beneficiaries is highlighted should demand outstrip supply, thereby prioritizing certain groups such as people already on ART, pregnant women and young children. The ART equity policy also underlines the need for ART to be delivered through mechanisms which will not exclude the poor, within a comprehensive response for HIV and AIDS care and in a way that does not take away resources from the other essential health services. ART is provided within a 'public health approach' which involves providing a fixed-dose combination treatment to eligible patients using a guardian supported programme [15]. The programme has a standardized system for patient recruitment, follow up, registration and monitoring and reporting of treatment outcomes [15].

### Access to Counselling and Testing Services: The national picture

There has been a rapid increase in the number of sites offering CT as well as the number of people tested for HIV over the past few years. CT services are available in all the districts in Malawi. While there were only 70 CT sites in 2002, by the year 2006, the number of CT sites had increased to 351 static CT sites [16]. There is an urban bias in the availability of CT sites, for example in 2005, 48% of the sites were located in the rural areas though more than 80% of Malawians reside in rural areas[17]. However the static sites are supplemented by outreach and mobile clinics. The number of people tested from other programmes such as Prevention of Mother to Child Transmission (PMTCT) and Tuberculosis is increasing and this indicates some integration of CT into these programmes. Nevertheless, extra effort is required in scaling up PMTCT services. Approximately 98, 000 women giving birth every year are in need of PMTCT services [16]. However, as of 2005, only 40 Health facilities provided PMTCT services and only 9% of women giving birth had been tested for HIV. In 2006, the number of HIV tests increased to 661, 400 with a positivity rate of 20%. Among pregnant women attending ANC services, HIV testing more than doubled to 137, 996 tests when compared to the previous year when only 52,904 tests were conducted. It should also be noted that though important, the number of people tested for Sexually Transmitted Infections (STI) clinics is not included. Studies show that there have been challenges in providing CT services in STI clinics as CT counselors are not deployed to STI clinics and secondly, STI clinicians and nurses do not possess adequate time and skills to counsel and test STI patients for HIV resulting in low uptake of CT services among STI patients [18] (Table 1).

**Table 1:** Table showing trend of CT uptake from 2002–2006

	**2002**	**2003**	**2004**	**2005**
Number of HIV Testing and Counseling Sites	70	118	146	249

Number of Blood Donors tested	57,850	60,561	62,396	58,152
Number (%) tested who were HIV+	8,474 (15%)	9,180 (15%)	8,098 (13%)	6,218 (11%)

Number of ANC women tested	5,059	26,791	43,345	52,904
Number (%) tested who were HIV+	840 (17%)	3,383 (13%)	6,069 (14%)	7,052 (13%)

Number of persons tested at MACRO Sites	51,224	48,333	48,527	56,860
Number (%) tested who were HIV+	7,684 (15%)	6,794 (14%)	7,046 (15%)	7,371 (13%)

Number of patients/clients at health facilities and NGOs [MACRO included] who were HIV tested	35,407	79,584	129,199	314,448
Number (%) tested who were HIV +	16,305 (46%)	30,758 (39%)	43,422 (34%)	90,512 (25%)

**Total number of tests done:**	**149,540**	**215,269**	**283,467**	**482,364**
Number (%) tested who were HIV positive	**33,303** **(22%)**	**50,115 (23%)**	**64,635 (23%)**	**111,153 (23%)**

Source: MoH et al 2007, HIV and AIDS Services Situational Analysis Report 2007

### Access to ART: the national picture

ART has been provided free since June 2004 and scale up of ART provision is gathering pace. By end December 2006, there were 85,168 patients who had ever started on ART in both the public and private health sector [19]. 39% of the patients were male while 61% were female. The majority of patients were adults, and 7% were children, aged 14 years or below. The private sector contributed about 4% of all patients who had ever accessed ART. In 2006, according to estimates based on the Antenatal Clinic sentinel surveillance projections, it was estimated that a total of 196,076 patients were in need of ART [20]. This means that only 43% of all patients who were in need of ART had accessed the treatment.

Malawi has set up its ART universal access target reaching 50% of those who are in need of treatment. In the year 2006, the goal for ART scale up was to put 35, 000 more patients on ART. This goal was achieved and was surpassed as a total of 45, 351 patients were started on ART during this year [19]. Though it is commendable that targets were met, the question needs to be asked whether the targets where challenging enough: there is clearly still unmet need.

While in the public sector ART is provided free at the point of delivery, in the private sector patients pay a heavily subsidized fee of about $3.6 per month. In addition to this, patients have to pay consultation costs and the costs of other drugs they may need. Access through the private sector means that patients are more likely to receive fuller laboratory assessments, for example as of September 2006, only 11% of the patients had access to baseline CD4 count as compared to 32% in the private sector [21]. WHO clinical staging is mainly used for determining eligibility for ART in the public sector as CD4 machines are only available in a few central and district hospitals.

The rationale behind the MoH working collaboratively with the private sector is that this will take the pressure off the public sector where human resources are limited and ideally enable more poor people to access ART from public facilities [22]. A proposal to formalise relationships between MoH and private sector to co-ordinate private sector delivery of ART was been finalized and adopted [22]. This lays out the modalities of ARV delivery by the private sector, which includes provision of ART at a subsidized cost and involvement of private sector in monitoring and evaluation of the ART programme. As of 2006, the private sector was contributing 4% of the total number who had ever started ART [19].

### CT and ART access by gender, age, geography and socio-economic status

Not all health facilities consistently recorded sex in the years preceding 2005. However, insights can be gained from a few facilities. In 2004, approximately equal numbers of males 37, 302 (50%) and females 37, 405 (50%) accessed CT services from 56 integrated health facilities [17]. However, when women who also access CT through the Prevention of Mother to Child Transmission programme (totaling 43, 345) are included, more women than men accessed CT services in 2004. Data from an NGO providing CT shows a different picture: in 2004 more males 33, 441 (69%) than females 15, 086 (31%) accessed CT services provided by the Malawi Counseling and Resource Organisation (MACRO) [23].

National CT registers were revised in 2006 and sex and age specific data is now recorded. Out of the 661, 400 HIV tests conducted in 2006, 289, 000 (44%) were with males while 372, 400 (56%) were with females. The Situational Analysis of HIV and AIDS services in 2006, collated data from 154 sites for a total of 78,224 testing encounters between October and December, 2006 [16]. Out of these, 28,192 (36%) were with males whilst 50, 032 (64%) were with females. Gender differences were more pronounced amongst adults aged 15 years and above than amongst children. Amongst children below 15 years, 47% were male and 53% female; for those over 15 years of age only 35% were male. These differences are probably largely explained through women's access through programmes supporting the prevention of mother to child transmission. These programmes are an important entry point to CT for rural and urban women. Further supporting women's access to CT through PMTCT and other avenues is important given the stark gender differences in prevalence, especially amongst younger groups. The HIV and AIDS prevalence among females in the age group (15–24 years) is reported to be four to six times higher than amongst males in the same age group [24].

The data shows that men are being left behind in accessing CT, HIV prevention and treatment interventions, and this needs to be addressed. The Malawi Demographic and Health Survey for 2004, also showed that more women (12.5%) reported to have accessed HIV testing and received their results than men (11.7%) [25]. Analysis of data of patients accessing ART from the Lighthouse Clinic in Lilongwe, shows that men generally tend to access ART when they are sicker and their CD4 levels are lower than women and therefore also tend to have poorer treatment outcomes [26].

The Malawi Demographic and Health Survey in 2004 also showed that people from the urban areas are more likely to report HIV testing than those from the rural areas, similarly those with higher primary school education levels and those in higher wealth quintiles are more likely to test for HIV than those in the lower wealth quintiles.

Since CT is the entry point to accessing treatment, it is important that factors that encourage and prohibit access to CT are addressed – amongst women who have higher HIV prevalence rates and amongst men who have lower access and uptake of services. It is also necessary to encourage more stand alone CT sites, as of end 2006 there were only five such sites available in the country.

As with counselling and testing, there are also more females (61%) than males (39%) accessing ART [19]. Even when HIV prevalence amongst males and females is taken into account more women than men are accessing ART, as shown in Table 2.

**Table 2:** Proportion of males and females accessing ART in Malawi

Projected population^i^		HIV prevalence^ii^		Estimated Infected population^iii^		Number ever accessed ART^iv^		Proportion on treatment	
Male	Female	Male	Female	Male	Female	Male	Female	Male	Female

6,275,533	6,482,350	10%	13%	627,553	648,235	33,617	51,551	5.2%	8.2%

^i^National Statistical Office projected population based on the 1998 Malawi Population and Housing Census

^ii^Malawi Demographic and Health Survey 2004

^iii^Proportion calculated from projected population and the HIV prevalence

^iv^MoH report on patients accessing ART, MoH 2007

Analysis by age and gender in 5 districts in Malawi showed that in the younger age group (0–12 yrs) access is similar between males and females. However, from the age of 13 more females than males access ART, particularly amongst those aged 25–34, which could reflect higher female sero-positivity in this age group [27].

About 7% (5909) of all the patients accessing ART in 2006 were children under the age of 15. It is not clear whether children proportionately access ART more or less when compared to their adult counterparts as there are no population based surveys that estimate HIV prevalence amongst children. However, there are particular challenges in the delivery of ART to children which include the need for specialized health workers and the longer client -health provider interactions that can be necessary due to the need for more thorough clinical investigations [28]. These need further investment as do prevention of mother to child transmission programmes.

### Towards promoting equity in CT and ART provision

Malawi has made good progress in rolling out CT and ART. The following three inter-related barriers need action to promote equity in access to CT and ART and adherence to ART: (1) human resources, (2) further integration between services and (3) promoting further accessibility amongst poor and marginalized groups.

### Strengthening Human Resources for Health

CT and ART provision brings new demands to already overstretched health staff and has the potential to divert resources and attention away from other services within the Essential Health Package. In Malawi, the biggest challenge for expanding ART expansion is the human resource shortage [15,29]. There are severe staff shortages in Malawi with an average vacancy rate of around 50 percent for all professional health workers posts sector-wide [30]. The total number of established posts in the health sector comprising the Ministry of Health and the Christian Health Association of Malawi as of 2005 was 28,600. Of these, 72% were filled, leaving 10,800 (38%) vacant, mainly across service providers/professional health worker categories. These vacancy rates pose serious challenges to the equitable and sustainable delivery of ART in Malawi. Using the estimated numbers of health professionals derived from the international literature, Muula et al calculated that ART delivery in Malawi in 2007 would use up 15.7 – 31.4% of all physicians and clinical officers, 66.5% – 199.3% of all pharmacists and pharmacy technicians and 2.6–9.2% of all available nurses [31].

The human resource crisis in the public sector [32,33] is one of the key reasons behind the explicit decision in Malawi to collaborate with the private health sector in the delivery of ART, as further overloading of the public sector could be detrimental. As the number of patients is increasing there is also an increasing demand for larger numbers of health workers in the ART programme. Further strengthening of human resources for health is critical. Harries et al stress the importance of better HR management, training, retention, motivation and supporting health worker access to ART [34].

### Strengthening integration between CT/ART services and other related health services

Further integration of both CT and ART services with other health services is critical given the human resource constraints and the importance from an equity perspective of spreading the benefit from the new resources being made available for ART expansion. This would allow for strengthening rather than undermining of the broader health services. Service integration means that services are more likely to be available to those in need too: such as TB patients and pregnant women who are underrepresented in treatment populations [15]. Statistics show that by end of 2006, only 8 percent of women ever started on ART as a result of referral from prevention of mother to child prevention programmes. There are missed opportunities too in integration with tuberculosis services, especially given HIV and tuberculosis co-infection rates of more than 70% [35]. Data up to September 2006 indicates that only 17 percent of all patients started on ART were patients with active or previous tuberculosis [19]. Even though tuberculosis and HIV co-infection is high there is a lack of horizontal integration in the service delivery of the two programmes. The tuberculosis and ART programmes are parallel vertical programs with different structural arrangements such that HIV positive tuberculosis patients who are on ART separately visit the TB clinic to collect anti-tuberculosis drugs and then the ARV clinic to collect ARV drugs. This has a negative impact on the patients who are required to report to two separate clinics on separate days. The impact on patients includes incurring of direct costs in form of transport and food and also opportunity costs of accessing the services [9]. It is reported that patients sometimes miss their appointments to get treatment as it becomes costly for them to make two separate visits for each appointment especially with long waiting times [9].

### Supporting further equity analysis and access to services by different groups

The decision to provide ART *free *on a first come-first served basis since 2004 is an extremely positive step from an equity perspective and is to be commended. Studies conducted prior to 2004 when patients were charged for drugs revealed exceptionally high levels of drop out [36]. Qualitative work conducted by the REACH Trust with female and male patients and caregivers of children on ART at the Lighthouse Clinic in the capital, Lilongwe revealed that cost was the key barrier to both access and adherence during the time when patients had to pay for CD4 counts and ART [37]. Since ART has been provided free, the Lighthouse clinic in the Capital Lilongwe, has witnessed more women, younger patients and those at an earlier stage of immuno-suppression starting ART, indicating that these groups may have faced cost challenges at the time when drugs were provided at cost [37].

However, despite free service provision in public health facilities women, men, boys and girls can still face a complex range of barriers, costs and opportunity costs in accessing CT, ART and adhering to ART as has been evidenced in access to TB services [38,39] which are provided for free. A qualitative study conducted by the REACH Trust in Thyolo 2005 (when ART was provided free), revealed the following key barriers to access and adherence for ART [40,41]

• Transport costs for the patient and the guardian for the patient's lifetime as well as livelihood costs including food.

• Lack of social support.

• Requirement of a guardian.

• Stigma/discrimination.

• Changes in health status.

• Long waiting time at the hospital.

Of all the reasons given, lack of food and transport costs were most commonly cited by the respondents. They pointed out that taking the drugs without food makes one to feel very dizzy (*kupanga chizumbuzumbu*). Due to their illness the patients are unable to find their own food either through buying or cultivating. The challenges posed by transport are illustrated in the following quote:

*"Imagine this is just my first month but I'm already tired. I'm supposed to foot transport costs for two people whenever we come here. Now I wonder that if things will continue to be like this in future am I going to adhere to the drugs? I'm saying this based on the instructions attached to these drugs -that one has to take them for life, without skipping scheduled times. Thus one may fail to adhere to the drug due to transport costs." *(In-depth interview with a 30 year old craftsman, Thyolo).

These costs and opportunity costs are exacerbated as ART and CT services are more concentrated in the urban areas and at district and tertiary levels of health provision. Long waiting times at the health facilities have been witnessed as patients especially from the rural areas have to travel long distance and spend money on food, and in some instances spend nights away from home so that they can receive treatment [40,42].

To address these costs and opportunity costs there is a need to develop innovative approaches to decentralizing services and bringing them closer to poor rural women, men, boys and girls. This is very challenging given the human resource constraints and the need to maintain quality services and promote adherence. There is need for new approaches such as increased roles for Health Surveillance Assistants and community groups in patient education and adherence support. These increased roles should be supported by adequate training.

## Conclusion

Malawi is arguably unique in having an Equity in Access to ART policy. This and discussions around equity in key CT documents reflects the priority given to equity by key stakeholders in Malawi. Considering the limited financial, human and infrastructural resources available, Malawi has made tremendous strides in the provision of CT and ART. However, many challenges remain.

There is an urban bias in the provision of CT and ART services which means that uptake of both of these vital services is relatively low in rural areas. There are many people in need of ART who are not yet accessing ART. Constraints to access and adherence relate to (1) human resource and infrastructural constraints and (2) the costs (e.g. transport, food) and opportunity costs (e.g. missing work) of accessing and adhering to services. Ability to overcome these costs is shaped by poverty, gender and geography. There is limited information on adherence to ART and this is critical from an equity perspective. Equity monitoring is important, including age and sex in CT and ART registers is an important step forward, simple measures to assess socio-economic status would also be beneficial. Ongoing equity monitoring and adaptation of services to meet the needs of poor women, men, girls and boys is critical to ensure that equity in CT and ART provision becomes a reality on the ground and not just an admirable sentiment in policy documents. There is a need to move beyond free on a first come first served basis and explicitly work to address the barriers poor women and men face in accessing free services.

Key recommendations towards this are as follows:

### Recommendations

#### Across the health sector

1. Maintaining, motivating and training health workers, with a particular emphasis on rural areas.

2. Supporting integration between tuberculosis and CT and ART services.

3. Strengthening prevention of mother to child transmission programmes to enhance women's access to CT & ART.

#### For CT

4. Increasing CT provision and human resources for CT particularly in rural areas.

5. An explicit strategy to maximize access of different groups, such as men – including analysis of the distribution of different types of sites – integrated and stand alone.

#### For ART

6. Further decentralisation of ART services to increase access amongst the rural poor.

7. Further investment and innovation in supporting children's access to ART.

8. Further discussion of human resources for ART with possible increased roles for Health Surveillance Assistants and community groups in patient education and adherence support, and development of training and support packages to equip these groups with appropriate skills.

9. Ongoing equity monitoring to develop strategies to encourage groups that are under-utilising services.

## Competing interests

The authors declare that they have no competing interests.

## Authors' contributions

All authors contributed to an initial workshop to discuss key resources available. All authors commented on the emerging drafts of the paper. LN, GB and TB conducted follow up visits with CT and ART providers to gather additional information.
